# An Interaction Network of the Human SEPT9 Established by Quantitative Mass Spectrometry

**DOI:** 10.1534/g3.119.400197

**Published:** 2019-04-11

**Authors:** Matthias Hecht, Reinhild Rösler, Sebastian Wiese, Nils Johnsson, Thomas Gronemeyer

**Affiliations:** * Institute of Molecular Genetics and Cell Biology, Ulm University, 89081 Ulm, Germany; † Core Unit Mass Spectrometry and Proteomics, Ulm University, 89081 Ulm, Germany

**Keywords:** proteomics, quantitative mass spectrometry, septins, interaction map

## Abstract

Septins regulate the organization of the actin cytoskeleton, vesicle transport and fusion, chromosome alignment and segregation, and cytokinesis in mammalian cells. SEPT9 is part of the core septin hetero-octamer in human cells which is composed of SEPT2, SEPT6, SEPT7, and SEPT9. SEPT9 has been linked to a variety of intracellular functions as well as to diseases and diverse types of cancer. A targeted high-throughput approach to systematically identify the interaction partners of SEPT9 has not yet been performed. We applied a quantitative proteomics approach to establish an interactome of SEPT9 in human fibroblast cells. Among the newly identified interaction partners were members of the myosin family and LIM domain containing proteins. Fluorescence microscopy of SEPT9 and its interaction partners provides additional evidence that SEPT9 might participate in vesicle transport from and to the plasma membrane as well as in the attachment of actin stress fibers to cellular adhesions.

The septins are often named the fourth component of the cytoskeleton and gained growing attention over the past few years (Menon and Gaestel 2017).

The mammalian genome encodes thirteen different septins (SEPT1-SEPT12, SEPT14) (Russell and Hall 2011), of which SEPT2, SEPT7 and SEPT9 are nearly ubiquitously expressed, while SEPT1, SEPT3, SEPT12, and SEPT14 are tissue specific. The remaining septins are expressed widely, but are not present in all tissues (Dolat *et al*. 2014). Based on sequence homology all septin subunits can be sorted into one of the four subgroups SEPT2, SEPT3, SEPT6 or SEPT7 (Neubauer and Zieger 2017).

In mammalian cells, septins regulate the organization of the cytoskeleton, vesicle transport and fusion, chromosome alignment and segregation, and cytokinesis (Surka *et al.* 2002; Estey *et al.* 2010; Bowen *et al.* 2011; Fuechtbauer *et al.* 2011; Tokhtaeva *et al.* 2015).

Septins cross-link and bend actin filaments into functional structures such as contractile rings in cytokinesis or stress fibers in filopodia and lamellipodia during cell migration (Mavrakis *et al.* 2014). Mammalian SEPT2/6/7/9 complexes also interact directly with microtubules (Bai *et al.* 2013).

The basic septin oligomer in mammalian cells is a hetero-octamer composed of the SEPT2, SEPT6, SEPT7, and SEPT9 at a stoichiometry of 2:2:2:2 (9-7-6-2-2-6-7-9).

Septin oligomers polymerize into higher ordered structures such as rings, filaments, and gauzes by end-to-end and lateral joining. The assembly of septins does not only depend on their biochemical properties and posttranslational modifications, but also on intracellular structures and interaction partners.

Given the range of cellular functions associated with septins, it is not surprising that a variety of diseases are linked to loss or gain of septin functions. Abnormalities in septin expression are linked to male infertility, hereditary neuralgic amyotrophy, neurodevelopmental disorders, Alzheimer, and Parkinson (Cerveira *et al*. 2011; Dolat *et al*. 2014; Rozenkrantz *et al.* 2016; Shen *et al.* 2017).

Especially SEPT9 has been linked to a variety of diseases and to diverse types of cancer including prostate, breast and colon cancer (Connolly *et al.* 2011b; Song and Li 2015; Gilad *et al.* 2015; Verdier-Pinard *et al.* 2017). SEPT9 expression is characterized by a number of splice variants. The SEPT9 locus contains 13 exons and encodes at least nine isoforms (according to NCBI Reference Sequences, Gene ID: 10801). SEPT9 has been shown to act as an oncogene, although tumor suppressive properties have been reported as well (Connolly *et al.* 2011a).

SEPT9 is linked to several intracellular processes. SEPT9 binds and bundles microtubules (Bai *et al.* 2013) and plays an important role in cytokinesis by mediating the localization of the vesicle-tethering exocyst complex to the midbody (Estey *et al.* 2010). It interacts furthermore with F-actin and functions as a stress fiber cross-linking protein (Smith *et al.* 2015). Proteins normally act in networks and phenotypic variations of an organism or a single cell often arise from alterations of these networks (Charloteaux *et al.* 2011). Thus, experimentally deciphering and understanding the interaction network of a protein often complements classic, systematic approaches (Breker and Schuldiner 2014). For the septins in general and SEPT9 in particular, no targeted approaches aiming at systematically deciphering interaction partners have been conducted.

We addressed this shortcoming by applying a quantitative proteomics approach to establish an interactome of SEPT9 in human fibroblast cells. We could identify new interaction partners of the myosin family and LIM domain containing proteins that are resident in cell-cell adhesions. The identities of the interaction partners provide evidence that SEPT9 might participate in vesicle transport from and to the plasma membrane as well as in the attachment of actin stress fibers to cellular adhesions.

## Materials and Methods

### Generation of the constructs

A pEGFP-C2 based expression plasmid for GFP-SEPT9_i1 was kindly provided by Elias Spiliotis (Drexel University, PA, USA). To generate a TAP-SEP9 construct, the GFP sequence was excised with the *Nhe*1 and *Bsr*G1 restriction sites and replaced by a sequence encoding for a N-terminal TAP tag which was amplified from the plasmid pBS1761 (Euroscarf) (primers ^5′^gcacgctagcatgataacttcgtatagcatac^3′^ and ^5′^gctgtgtacagcttatcgtcatcatcaagtg^3′^; restriction sites underlined).

A mCherry-SEPT9 fusion protein was generated by replacing the GFP sequence by the mfCherry tag which was amplified from an expression plasmid kindly provided by Helge Ewers (FU Berlin, Germany).

To generate a 6his fusion protein for expression in *E. coli*, SEPT9 was PCR amplified (primers ^5′^ccgaaggccagcacggccgaaaacctgtacttccagggtaagaagtcttactcaggagg^3′^ and ^5′^ctgtgggccaaaaaggccttatcacatctctggggcttctgg^3′^; restriction sites underlined) from the GFP expression plasmid and cloned into the in-house constructed expression plasmid pES allowing for the expression of N-terminal 6his fusion proteins in *E. coli*.

An EGFP-MYO6 expression plasmid was kindly provided by Hans-Peter Wollscheid (IMB, Mainz, Germany). A C-terminal tail fragment of MYO6 spanning amino acids 835-1294 was PCR amplified (primers ^5′^gctagggatccaaacctcgcattgatggtctg^3′^ and ^5′^ctaggaattccctactttaacagactctgcagc^3′^; restriction sites underlined) from this plasmid and cloned in the pGEX2T plasmid (GE Healthcare) yielding a N-terminal GST fusion protein for expression in *E. coli*. A C-terminal tail fragment of MYO1C spanning amino acids 809-1063 was PCR amplified (primers ^5′^gctagggatccctggaccatgtgcgcacc^3′^ and ^5′^gctagtcgacgttcaccgagaattcagccgtg^3′^; restriction sites underlined) from cDNA obtained from in house prepared 1306 fibroblast mRNA and cloned in the pGEX2T plasmid.

A pcDNA-FRT/TO-GFP-LMO7 plasmid was obtained from the MRC Protein Phosphorylation and Ubiquitinylation Unit of the University Dundee, Ireland.

### Cell culture, immunofluorescence and microscopy

Immortalized 1306 skin fibroblast cells (Gebara *et al.* 1987) were a kind gift of Sebastian Iben (Ulm University, Germany). The cells were cultivated in DMEM (Thermo Scientific) supplemented with 10% FCS (Thermo Scientific) under humidified 5% CO_2_ atmosphere. For SILAC, TAP-SEPT9_i1 expressing cells were grown in SILAC-DMEM (Thermo Scientific) supplemented with 10% dialyzed FBS, 0.398 mM L-arginine-^13^C_6_^15^N_4_ hydrochloride (Sigma) and 0.798 mM L-Lysine-^13^C_6_^15^N_2_ (Sigma).

Plasmids were transfected by standard calcium phosphate transfection. For the generation of stable cell lines, Geneticin (G418, Formedium) was added 48 h post-transfection at a concentration of 500 ng/µl. After selection, cells were maintained under 250 ng/µl G418. The successful expression of the tagged SEPT9 constructs was verified by Western blotting and immunofluorescence (IF) using anti-ProteinA (Sigma) and anti-SEPT9 (Sigma) antibodies.

Cells for IF were grown on sterile coverslips placed in a 6-well cell culture plate in 2 ml medium or in 8-well tissue culture chamber slides (Sarstedt) previously coated with Poly-L-Lysine. The cells were fixed in 3% Paraformaldehyde and permeabilized with 1% TX-100 in DPBS (Biochrom) for 5 min at room temperature. After blocking with 10% BSA, cells were incubated for 1 h at 37° with primary antibody followed by the respective secondary antibody. A referenced list of all used antibodies for IF including the applied dilution is provided in Supplementary Table 2.

Cover slips were mounted with fluorescent mounting medium (Dako) and observed using an Observer SD confocal microscope (Zeiss) equipped with 488 nm, 561 nm and 635 nm diode lasers, a 63-fold Plan-Apochromat objective with lens aperture 1.4 and an Evolve 512K EMCCD camera (Photometrics). Image processing was done with the acquisition software of the microscope (Zen Vers. 2 blue, Zeiss) and with ImageJ.

### Affinity purification of SEPT9 complexes

Affinity purification of SEPT9 complexes was performed separately for TAP-SEPT9 and GFP-SEPT9 in a “mixing after purification” (MAP) approach.

Cells were grown to 80% confluency in one 175 cm^2^ bottle. The medium was aspirated, the cells washed once with PBS and subsequently detached using a cell scraper. The detached cells were pelleted and ice-cold lysis buffer (50 mM HEPES pH 7.5, 150 mM NaCl, 0.2 mM EDTA, 0.1% Triton X100, 8.3 μM Antipain, 0.3 μM Aprotinin, 1 mM Benzamidine, 1 μM Bestatin, 10 μM Chymostatin, 1.5 μM Pepstatin A, 5 μM Leupeptin, 1 mM PMSF, 10 mM β-Glycerophosphate, 10 mM NaF, 1 mM Sodium Orthovanadate) was added. The volume of lysis buffer including protease inhibitors was adjusted dependent on the cell pellet volume (approximately double pellet volume). For complete lysis, cells were incubated on ice and vortexed for 10 s at highest settings every 5 min for a total of 30 min followed by centrifugation at 13,000 × g for 10 min at 4°. The supernatant containing the soluble cytoplasmic and nucleoplasmic cellular proteins was directly used for separation by SDS-PAGE and Western blot analysis or affinity purification. Isotonic lysis buffers containing low concentrations of non-ionic detergent solubilize all extra- and intracellular membranes while cytoskeletal and nuclear structures remain intact and can be sedimented.

After cell lysis and determination of the protein concentration by a Bradford assay, the extract was adjusted to 10% Glycerol and the concentrations of the extracts were balanced by dilution of the higher concentrated extract with buffer. CNBr activated Sepharose (GE Healthcare) was coupled with human IgG (MP Biomedicals) according to the manufacturer’s protocol and stored as slurry in PBS/10 mM NaN_3_. The resulting hsIgG-Sepharose was equilibrated with lysis buffer and added to the cell lysates (15 µl slurry per mg of protein). Incubation was performed by rotating with 5 rpm overnight at 4° in an overhead shaker. The following day, another equivalent volume of hsIgG-Sepharose was added and incubated for additional 2 h at 4° while rotating at 5 rpm. The sepharose was pelleted at 100 × g for 3 min at 4° and transferred into Mobicol “F” columns (Mobitec) equipped with a 35 µm filter. After washing the slurry with 50-fold volume TAP buffer (20 mM Tris pH 7.5, 80 mM NaCl, 10% Glycerol, 1 mM DTT, 8.3 μM Antipain, 1 μM Bestatin, 10 μM Chymostatin, 1.5 μM Pepstatin A, 5 μM Leupeptin, 1 mM PMSF, 10 mM β-Glycerophosphate, 1 mM Sodium Orthovanadate), the columns were locked and two slurry volumes of TAP buffer was added. Proteins were eluted from the matrix by boiling in 2x Laemmli-buffer for 2 min at 95° and equal volumes of both sample and control were mixed for the MAP approach. The samples were jointly separated by SDS-PAGE on 6–12% BOLT Bis-Tris gradient gels (Thermo Scientific). The gel was stained by colloidal Coomassie by pre-incubation in staining solution (34% (v/v) methanol, 2% (v/v) phosphoric acid, 17% (w/v) ammonium sulfate) for 1 h at RT and subsequent addition of 0.066% (w/v) Coomassie brilliant blue G-250. After incubation overnight, the gel was de-stained with ddH_2_O and subjected to MS analysis. Affinity purification was performed as triplicate.

### Mass spectrometry

Each SDS-PAGE gel was cut into 14 pieces. Individual pieces were washed thrice by alternating incubation in 50 mM ammonium bicarbonate and 25mM ammonium bicarbonate / 50% Acetonitrile (ACN) for 10 min each. Following vacuum drying, samples were reduced with 5 mM DTT for 20 min at RT and subsequently alkylated with iodoacetamide for 20 min at 37°. After a second vacuum drying step, proteins were subjected to tryptic digestion overnight at 37° as described elsewhere (Wiese *et al.* 2007). Peptides were extracted in two rounds by adding 20 µl 0.1% Trifluoroacetic acid (TFA) / 50% ACN and incubation in an ultrasonic bath for 10 min each. ACN was evaporated and samples filled to 15 µl with 0.1% TFA.

Samples were measured using an LTQ Orbitrap Velos Pro system (Thermo Fisher Scientific) online coupled to an U3000 RSLCnano (Thermo Fisher Scientific) as described previously (Mohr *et al.* 2015), with the following modifications: Separation was carried out using a binary solvent gradient consisting of solvent A (0.1% FA) and solvent B (86% ACN, 0.1% FA). The column was initially equilibrated in 5% B. In a first elution step, the percentage of B was raised from 5 to 15% in 5 min, followed by an increase from 15 to 40% B in 30 min. The column was washed with 95% B for 4 min and re-equilibrated with 5% B for 25 min.

Database search was performed using MaxQuant Vers. 1.5.2.8 (www.maxquant.org) (Cox and Mann 2008). For peptide identification, MS/MS spectra were correlated with the UniProt human reference proteome set (www.uniprot.org) supplemented with the sequence of the TAP-SEPT9 construct, employing the build-in Andromeda search engine (Cox *et al.* 2011). The respective SILAC amino acids were selected, while Carbamidomethylated cysteine was considered as a fixed modification along with oxidation (M), and acetylated protein N-termini as variable modifications. False discovery rates were set on both, peptide and protein level, to 0.01. MaxQuant normalized ratios were used for statistical evaluation. If no ratio was calculated by MaxQuant, ratios were calculated manually in case overall intensity exceeded 1E6 to allow for quantitation of IP-specific proteins. For statistical evaluation, Significance B was calculated using Perseus 1.5.0.15 (www.maxquant.org) with default parameters.

The complete data evaluation results including statistics is provided in Supplementary Table 1.

### In vitro binding assays

6his-tagged SEPT9 was expressed in 250 ml LB medium at 37° for 3 h after addition of 0.1 mM IPTG at an OD_6oo_ of 1.0. The cell pellet was resuspended in 25 ml IMAC A (50 mM KH_2_PO_4_ pH 7.5, 300 mM NaCl, 20 mM Imidazole) containing e-complete protease inhibitor cocktail (Roche) and lysed by addition of lysozyme and ultrasound treatment.

The crude extract was subjected to IMAC chromatography using a 5 ml HisTrap excel column (GE Healthcare) and an ÄKTA Purifier chromatography system (GE Healthcare). The protein was eluted from the column in three consecutive elution steps of 15%, 40% and 100% IMAC B (50 mM KH_2_PO_4_ pH 8.0, 300 mM NaCl, 200 mM Imidazole). We considered approx. 70% purity after IMAC (judged by eye from a SDS-PAGE gel) as sufficient for further qualitative binding experiments.

The protein was transferred into PBS using a PD10 desalting column (GE Healthcare), concentrated and immediately used for binding assays.

GST-MYO6_835-1294_ and GST-MYO1C_809-1063_ were expressed in 50 ml SB medium at 18° for 6 h after addition of 0.1 mM IPTG at an OD_6oo_ of 0.6. Cell lysis was performed as described above in PBS and the GST fusion proteins were immobilized onto each 100 µl Glutathione Sepharose (GE Healthcare) from the crude extract. The beads were incubated with 2 µM 6his-SEPT9 for 1h and bound protein was detected after washing and elution from the beads by Western blot analysis using a mouse anti-6his primary antibody (Sigma). The eluted GST fusion proteins were detected using a mouse anti-GST primary antibody (Sigma).

### Data availability

The mass spectrometry proteomics raw data have been deposited to the ProteomeXchange Consortium via the PRIDE (Vizcaíno *et al.* 2016) partner repository with the dataset identifier PXD010773. Supplemental material available at FigShare: https://doi.org/10.25387/g3.7873481.

## Results and Discussion

SEPT9 has several splice isoforms. Isoform 1 (SEPT9_i1) contains all possible exons and is widely expressed whereas expression of several other isoforms is restricted to certain cell lines or tissues (Hall *et al.* 2005; Robertson *et al.* 2004). We consequently used SEPT9_i1 in all our experiments.

**Figure 1:**
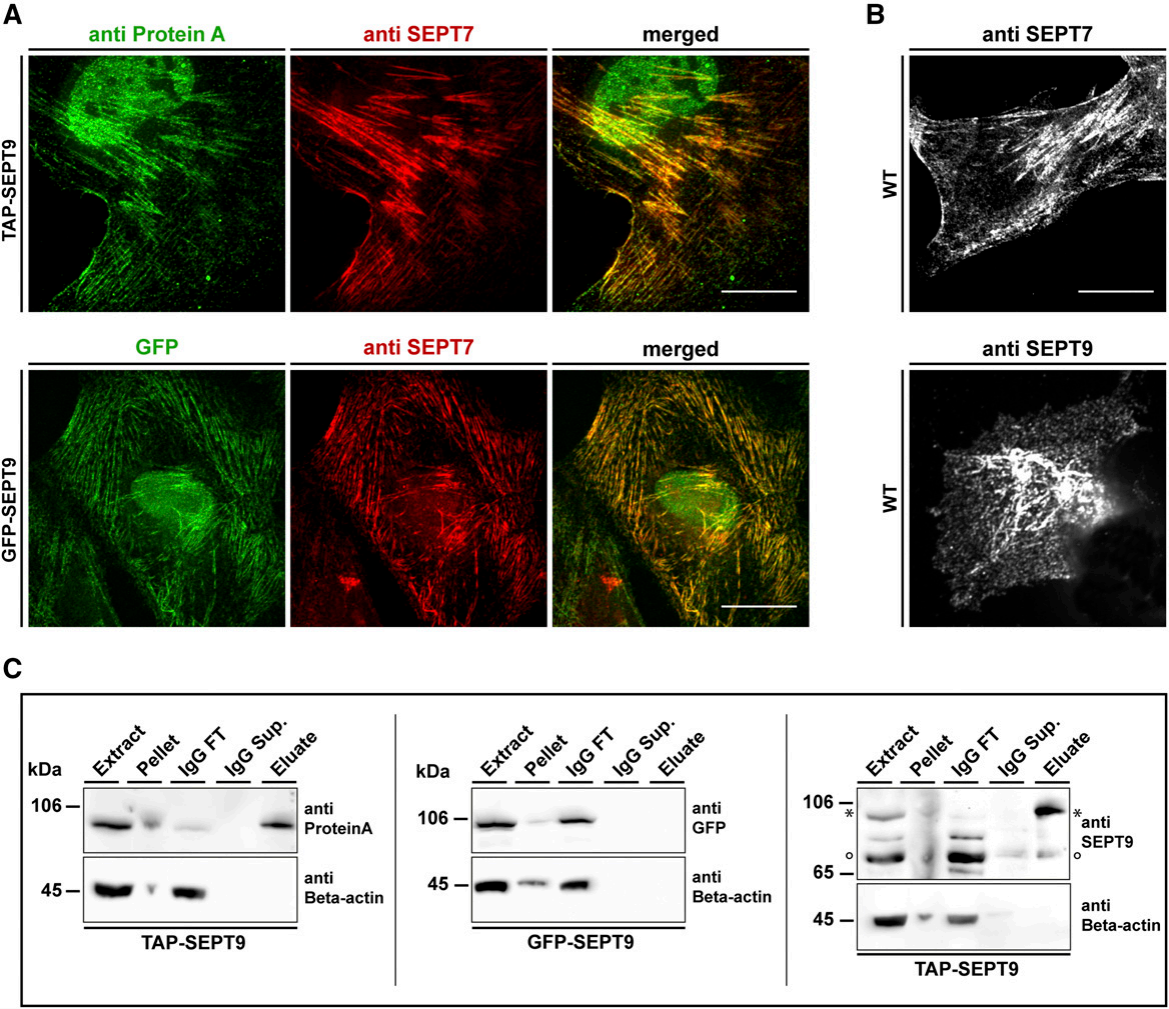
Cell lines and workflow for AP-MS of SEPT9 complexes. A) Characterization of the used cell lines. The colocalization of GFP-SEPT9 (upper panel) and TAP-SEPT9 (lower panel) with the endogenous septin cytoskeleton was probed by immunostaining of SEPT7. TAP-SEPT9 was visualized via an anti-ProteinA antibody. B) 1306 wild type fibroblasts were stained with anti SEPT9 and anti-SEPT7 antibodies. Scale bars represent 20 µm. C) Western blot analysis of the consecutive steps of the AP. Samples of the cell extract, pellet, supernatant of the beads after coupling (IgG sup.), washing step (IgG wash) and the eluate were separated by SDS-PAGE and Western blot was performed using an anti-ProteinA, anti-GFP or anti-SEPT9 antibody, respectively. The band marked with an asterisk represents TAP-SEPT9. The band marked with a circle represents the endogenous SEPT9.

To investigate the interactome of SEPT9_i1 in human 1306 fibroblast cells we applied an established SILAC-based workflow on cell lines expressing either TAP-SEPT9 or, as control, GFP-SEPT9 (Oeljeklaus *et al*., 2009; Renz *et al.* 2016). Both fusion proteins were expressed under the control of a CMV promoter. We confirmed by immunostaining of the endogenously expressed SEPT7 that constitutive over-expression of tagged SEPT9 does not interfere with the architecture of the septin cytoskeleton. Both GFP-SEPT9 and TAP-SEPT9 colocalized in filamentous structures with the septin cytoskeleton (Figure 1A and Suppl. Figure 1A). Cells overexpressing the SEPT9 constructs contain more filaments than non-transfected wild type cells (Figure 1A and 1B). SEPT9 occupies the terminal position of septin rods and has a stabilizing effect on septin fibers (Kim *et al.* 2011). The observed effect might thus result from stabilization of filaments due to the surplus of SEPT9. A fraction of the tagged SEPT9 was detected in the nucleus. This localization might represent an artifact of overexpression (Connolly *et al.* 2011b). Additionally, SEPT9_i1 contains a bipartite nuclear localization signal as judged by *in silico* prediction using PSORT. We used only the cytosolic protein fraction after cell lysis. This fraction might contain some of the originally nuclear SEPT9 and other nucleoplasmic proteins as a small amount of known nucleoplasmic proteins were also found in the cytosolic pool after cell lysis (Suppl. Figure 1B).

**Figure 2:**
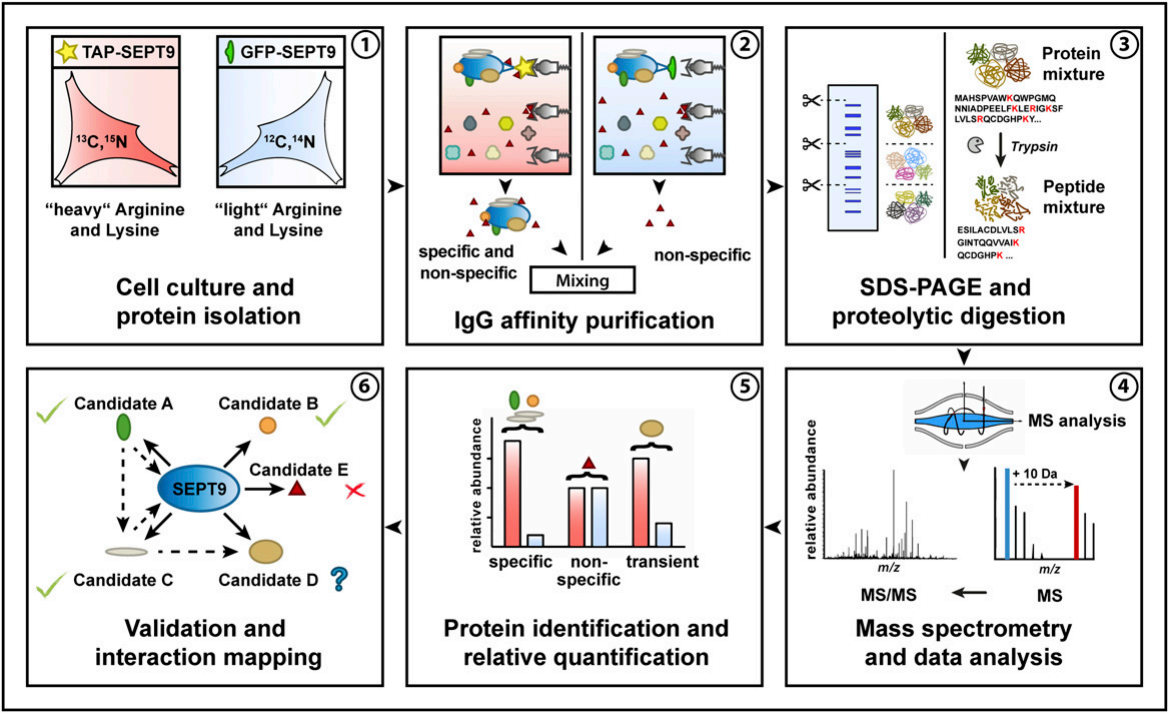
Workflow of the performed “mixing after purification” AP approach with subsequent MS analysis.

The TAP-SEPT9 expressing cell lines were labeled with heavy amino acids by cultivating them in L-^13^C_6_^15^N_4_ Arginine and ^3^C_6_^15^N_2_ L-Lysine containing medium (SILAC). The GFP-expressing cell lines were not labeled. For the identification of septin-interacting proteins, SEPT9 complexes were affinity-purified from the cytoplasmic fraction in a mixing after purification approach (MAP) (Oeljeklaus *et al*. 2009). Equal volumes of eluates were combined prior to SDS-PAGE and subsequent LC/MS analysis. Affinity purifications were monitored by Western blot analysis of representative samples taken during the purification process (Figure 1C). As expected, TAP-SEPT9 could be detected in the elution from the affinity matrix whereas GFP-SEPT9 and endogenous SEPT9 could not. Expression levels of tagged SEPT9 did not exceed the levels of endogenous SEPT9 or other endogenous septins (Figure 1C ans Suppl. Figure 1C). The whole AP-MS workflow is summarized in Figure 2.

We performed three independent AP-MS purifications. For each identified protein we calculated a “heavy over light” (H/L) ratio and significance B values (SigB) (Cox and Mann 2008). The LOG_2_(H/L) reflects the enrichment of a protein in the TAP-SEPT9 purification (H) in comparison to the GFP-SEPT9 purification (L).

SigB scores reflect the probability of a given protein ratio to deviate from the normal distributed ratios of all quantified proteins, by also taking into account the improved accuracy of proteomic quantifications for higher abundant proteins. We considered a protein in a replicate as significantly enriched at a LOG_2_(H/L)>2 and SigB values < 0.05.

Two groups of SEPT9 interacting proteins were initially defined: Class I interactors had a LOG_2_(H/L)>2, were significant according to SigB and were identified in at least two of the three replicates. Only 8 of the potential interaction partners fulfilled these criteria. Many *bona fide* interactors with a high LOG_2_(H/L) ratio were only identified in one replicate. We defined a second group of interactors with less stringent criteria compared to Class I. Class II interactors were either found in only one experiment with a LOG_2_(H/L)>2 and a significant SigB or had LOG2(H/L)>2 (but not a significant SigB) in at least two replicates. 28 candidates fell into this group. All proteins identified as Class I and Class II interactors including their LOG_2_(H/L)>2 ratios are summarized in Table 1.

**Table 1:** List of all identified specific SEPT9 interactors (Class I and Class II) and manually curated Class III candidates

UniProt ID	Protein name	Gene name	AP1 LOG_2_(H/L)	AP2 LOG_2_(H/L)	AP3 LOG_2_(H/L)
P08670	Vimentin	*VIM*	5.061	2.741	0.107
O15231	Zinc finger protein 185	*ZNF185*	5.600	2.710	1.282
A6NMH6	Septin-8	*SEPT8*	7.346	—	3.568
Q15019	Septin-2	*SEPT2*	2.167	2.595	1.856
E7ES33	Septin-7	*SEPT7*	2.639	4.887	1.778
D6RGI3	Septin-11	*SEPT11*	2.901	3.891	2.060
D6RGI3	Aminopeptidase N	*ANPEP*	3.029	2.185	—
Q8WWI1	LIM domain only protein 7	*LMO7*	—	2.225	3.794
E9PDF6	Unconventional myosin-Ib	*MYO1B*	4.846	2.220	0.623
E7EPZ9	Tenascin-X	*TNXB*	7.652	—	—
P17252	Protein kinase C alpha type	*PRKCA*	3.279	—	—
Q9UBI6	Guanine nucleotide-binding protein G(I)/G(S)/G(O) subunit gamma-12	*GNG12*	3.828	—	—
P10301	Ras-related protein R-Ras	*RRAS*	6.174	—	—
Q96FN4	Copine-2	*CPNE2*	3.924	—	—
E9PNW4	CD59 glycoprotein	*CD59*	5.288	—	0.635
Q6YHK3	CD109 antigen	*CD109*	6.287	—	—
Q6XZB0	Lipase member I	*LIPI*	3.524	—	—
P05362	Intercellular adhesion molecule 1	*ICAM1*	4.501	—	—
H3BNE1	Synaptosomal-associated protein 23	*SNAP23*	4.958	—	—
Q86WV6	Stimulator of interferon genes protein	*TMEM173*	4.491	—	—
O43707	Alpha-actinin-4	*ACTN4*	2.456	2.004	0.377
H0Y7A7	Calmodulin	*CALM2*	2.538	2.054	1.017
P08754	Guanine nucleotide-binding protein G(k) subunit alpha	*GNAI3*	3.005	—	—
F8W7R3	Fanconi anemia group I protein	*FANCI*	3.075	—	—
Q15283	Ras GTPase-activating protein 2	*RASA2*	3.116	—	—
Q9Y2F5	Little elongation complex subunit 1	*ICE1*	—	—	6.978
Q15746	Myosin light chain kinase	*MYLK*	—	4.389	1.663
Q9P2N5	RNA-binding protein 27	*RBM27*	—	6.739	0.250
Q9BX40	Protein LSM14 homolog B	*LSM14B*	—	3.080	1.798
Q9BX40	Protein LSM14 homolog A	*LSM14A*	—	3.078	1.797
Q03135	Caveolin-1;Caveolin	*CAV1*	1.941	3.644	—
E9PE29	Zinc finger protein 106	*ZNF106*	—	3.688	—
Q6NYC8	Phostensin	*PPP1R18*	—	6.145	—
Q8IYI6	Exocyst complex component 8	*EXOC8*	—	—	6.289
Q86UE4	Protein LYRIC	*MTDH*	—	3.969	—
P00505	Aspartate aminotransferase, mitochondrial	*GOT2*	—	—	3.541
F5H6E2	Unconventional myosin-Ic	*MYO1C*	4.519	1.450	0.357
F8W6L6	Myosin-10	*MYH10*	2.339	0.585	0.376
Q9UHB6	LIM domain and actin-binding protein 1	*LIMA1*	2.690	1.281	1.174
O14974	Protein phosphatase 1 regulatory subunit 12A	*PPP1R12A (MYPT1)*	2.987	0.130	0.486
Q6WCQ1	Myosin phosphatase Rho-interacting protein	*MPRIP*	3.061	0.159	0.560
E7EW20	Unconventional myosin-VI	*MYO6*	3.767	1.913	—

**Figure 3:**
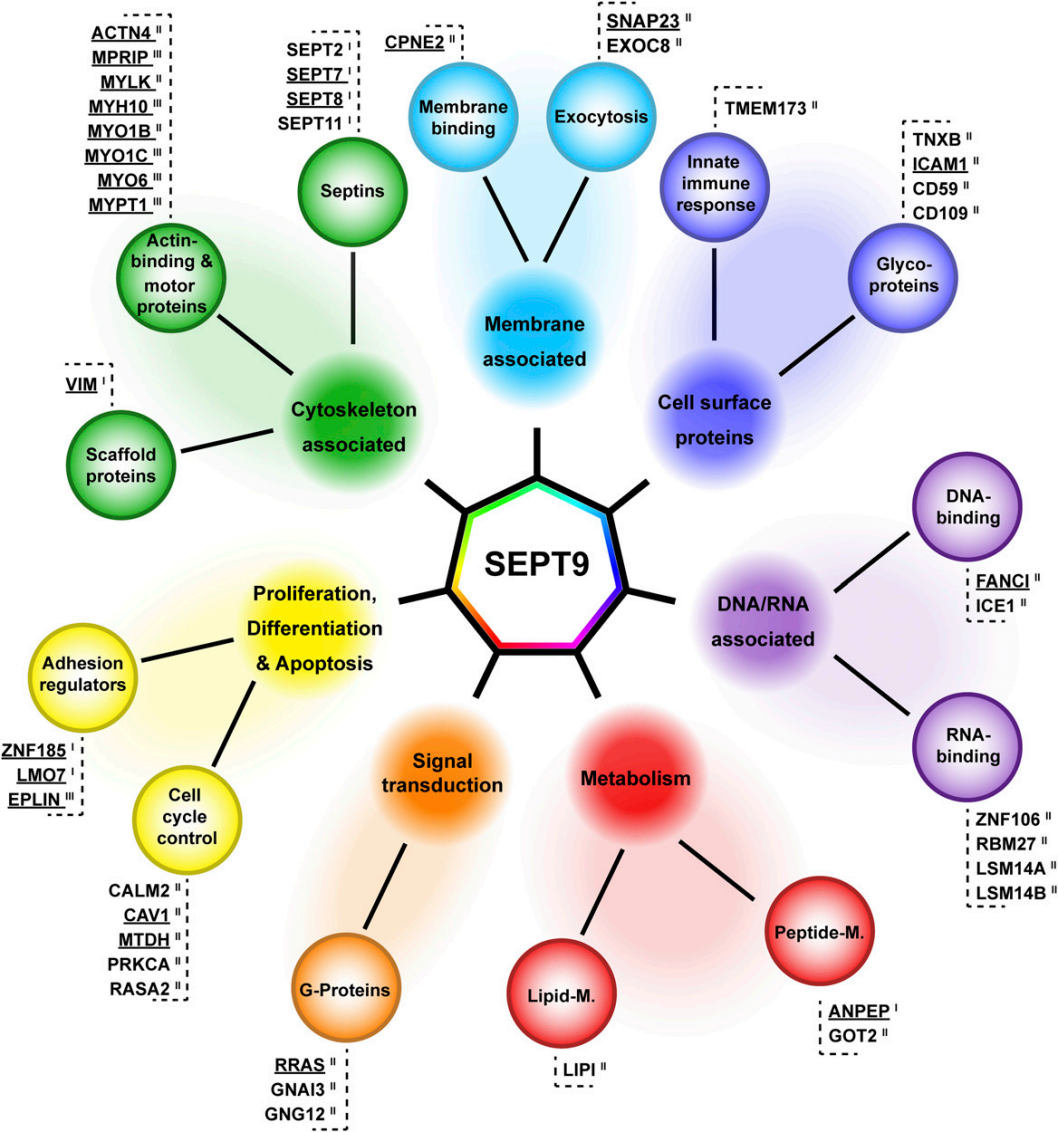
Interaction map of SEPT9 in 1306 fibroblast cells. Class I, II and III SEPT9 interactors identified by SILAC based AP-MS were categorized into seven functional groups, (i) cytoskeleton associated proteins, (ii) proteins involved in proliferation, differentiation and apoptosis, (iii) cell surface proteins, (iv) metabolic proteins, (v) signal transduction proteins, (vi) nucleic acid associated proteins and (vii) proteins associated with the plasma membrane. Candidates validated by IF are underlined.

We sorted the interaction partners according to their biological function into seven categories, namely (i) cytoskeletal and cytoskeleton associated proteins, (ii) proteins involved in proliferation, differentiation and apoptosis, (iii) cell surface proteins, (iv) proteins involved in metabolism, (v) signal transduction proteins, (vi) nucleic acid associated proteins and (vii) proteins associated with the plasma membrane. Based on these categories we draw a map of SEPT9 interactors (Figure 3).

SEPT2, SEPT7, SEPT8 and SEPT11 were found as Class I interaction partners of SEPT9. SEPT7 and SEPT2 are members of the canonical SEPT2-SEPT6-SEPT7 hexamer which is extended by SEPT9 as terminal subunit to an octamer. The interaction of SEPT9 with both subunits was previously shown by yeast two hybrid and affinity capture experiments, respectively (Nakahira *et al.* 2010; Sandrock *et al.* 2011). We did not detect SEPT6 as specific interactor, the remaining core subunit of the hexamer. However, the individual members within one septin subgroup can substitute for one another in the hexameric complex (Nakahira *et al.* 2010; Sandrock *et al.* 2011). As SEPT6 and SEPT8 belong to the same subgroup, we assume that SEPT8 is replacing SEPT6 in human 1306 fibroblast cells.

We subsequently selected a list of candidates for further investigation. First, we considered all Class I interactors. The intermediate filament Vimentin is highly expressed in fibroblasts and known to interact with a diverse set of other proteins inside the cell (Evans 1998). Aminopeptidase N (ANPEP, CD13) is an integral membrane protein with functions in cell adhesion and processing of bioactive peptides (Garay-Canales *et al*. 2018). It has a large extracellular domain and a short intracellular C-terminal tail. Among the Class I interactors were two LIM domain containing proteins, LMO7 and ZNF185. LIM domains contain tandem zinc-finger structures and function as a modular protein-binding interfaces (reviewed in te Velthuis *et al.* (2007)). Both proteins were shown to associate with actin structures and play a role in cancer progression (Tzeng *et al.* 2018; Zhang *et al*. 2007). One more LIM domain containing protein, LIMA1 (herein from now on referred to as EPLIN, the product of the *LIMA1* gene), was identified with a LOG_2_(H/L)>2 in one of the replicates, however below the SigB threshold. We decided to consider this protein for further validation and to compare its properties with the other two LIM proteins.

From the Class II interactors we picked representative candidates from each functional category (except the cell surface proteins) for validation (underlined proteins in Figure 3). We selected MYO1B and the myosin associated protein MYLK among the Class II candidates as SEPT9 was already shown to competes with myosin for binding to the same site on F-actin (Smith *et al.* 2015). However, a direct interaction of SEPT9 with myosin motors has not been shown yet. Another septin, SEPT2, has been previously reported to interact directly with non-muscle myosin II (Joo *et al*. 2007) and thereby enables myosin II activity in interphase and dividing cells.

We screened our data set for further myosin or myosin associated proteins. We identified five proteins - MYO1C, MPRIP, MYH10, PPP1R12A (MYPT1) and MYO6 - with a LOG_2_(H/L)>2. However, these hits were identified each in only one replicate as specific SEPT9 interactor and they were not significant according to SigB. Although each of these proteins does not meet our (unbiased) selection criteria, they present together the largest functional group in our dataset. Consequently, we grouped these myosins and myosin associated proteins together with EPLIN into the category of the manually curated Class III interactors (Table 1) and evaluated these proteins also for interaction with SEPT9.

Altogether, we selected Vimentin, ANPEP, EPLIN, ZNF185, LMO7, SNAP23, MYPT1, MPRIP, MYO1B, MYO1C, MYO6, MYH10, MYLK, ACTN4, CAV1, FANCI and RRAS for validation experiments.

**Figure 4:**
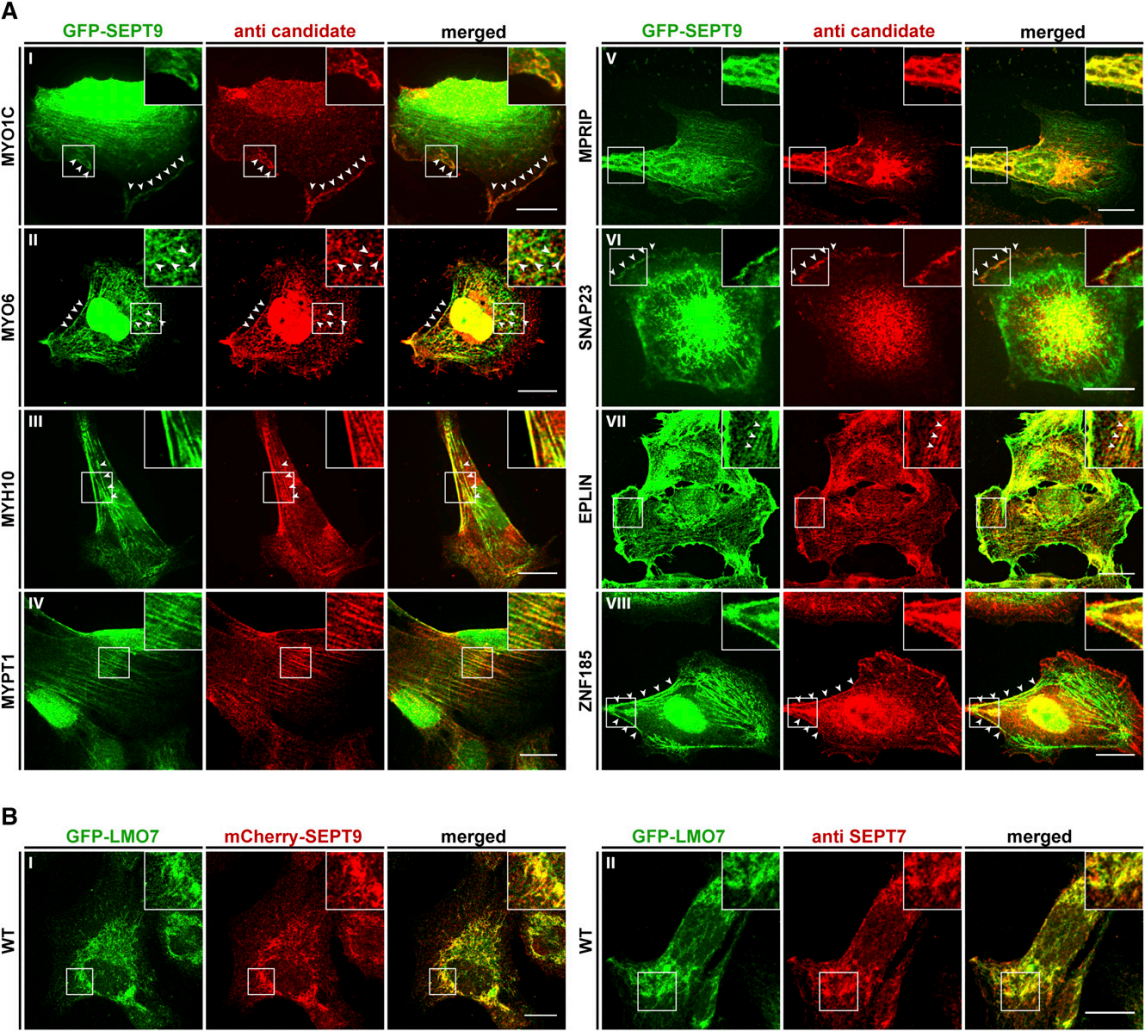
Colocalization of interaction partners with SEPT9 in GFP-SEPT9 expressing cells. A) The indicated candidate proteins were immunostained with a suitable primary antibody, followed by an Alexa555 coupled secondary antibody. GFP-SEPT9 was observed directly via its GFP. White arrowheads mark colocalizing structures. B) Colocalization of GFP-LMO7 with mCherry-SEPT9 in transiently transfected 1306 cells (left panel) and colocalization with the endogenous septin cytoskeleton by IF via an anti-SEPT7 antibody (right panel). Images were assembled from z-projections. The scale bars represent 20 µM.

**Figure 5:**
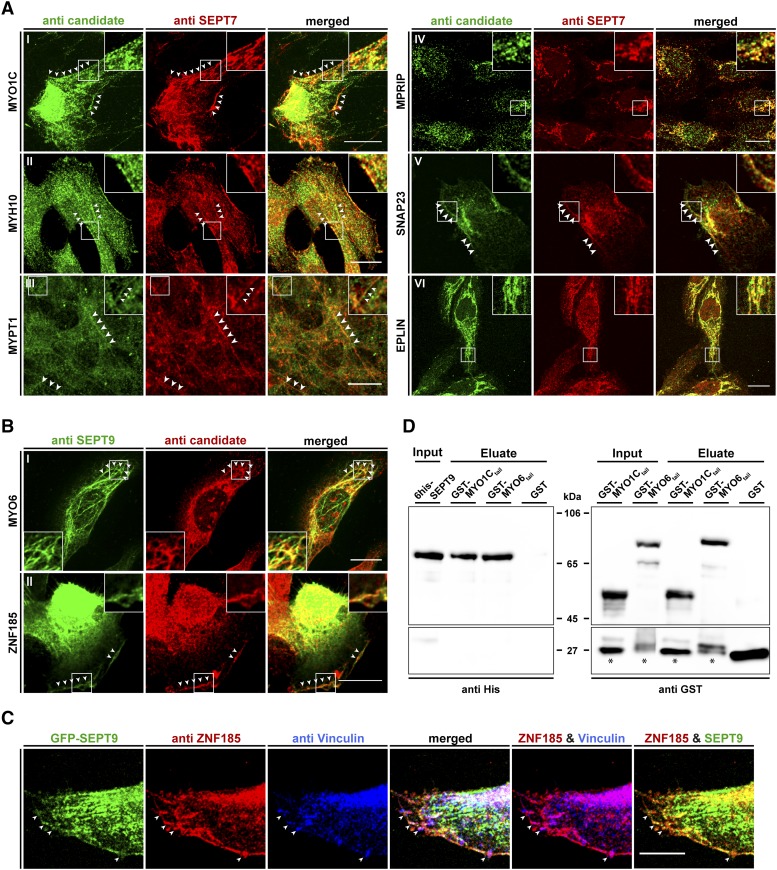
Colocalization of interaction partners with the septin cytoskeleton in 1306 fibroblast cells and pulldown analysis showing a direct interaction of SEPT9 with myosin motors. A) The indicated candidate proteins were immunostained with a suitable primary antibody, followed by an Alexa488 coupled secondary antibody. The endogenous septin cytoskeleton was immunostained by an anti-SEPT7 primary antibody followed by an Alexa555 coupled secondary antibody. White arrowheads mark colocalizing structures. B) Colocalization of MYO6 and ZNF185 with the endogenous septin cytoskeleton by immunostaining of SEPT9 with an anti-SEPT9 primary antibody followed by an Atto488 coupled secondary antibody. Due to species incompatibility of the available primary antibodies against MYO6 and ZNF185, these two candidates had to be visualized via an Alexa555 coupled secondary antibody. White arrowheads mark colocalizing structures. C) Colocalization of ZNF185, Vinculin and SEPT9 in GFP-SEPT9 expressing cells. ZNF185 was visualized as in figure part B and Vinculin was visualized by an antibody directly coupled to Alexa647. White arrowheads mark sites of focal adhesions. Images in parts A-C were assembled from z-projections. The scale bars represent 20 µM. D) *In vitro* pulldown experiment showing direct binding of 6his-SEPT9 to tail fragments of myosin motors. GST-MYO6_835-1294_, GST-MYO1C_809-1063_ and free GST as control were immobilized on Glutathione sepharose and incubated with purified 6his-SEPT9. Samples of the SEPT9 input, the GST-fusion protein input and the eluates from the beads were subjected to SDS-PAGE and subsequent Western blot analysis using anti-his or anti-GST primary antibodies, respectively. Bands marked with an asterisk represent a GST containing degradation product of the GST-myosin fusion proteins.

We performed co-localization studies by immunofluorescence (IF) in the stably GFP-SEPT9 expressing cell line (Figure 4) and in wildtype fibroblasts using an anti-SEPT7 antibody (Figure 5). The results were consistent in both cell lines for all tested candidates.

For some candidate proteins (ANPEP, LMO7, MYLK, ACTN4, CAV1, MYO1B), the respective antibodies were too unspecific to draw any conclusions (data not shown). Vimentin and FANCI did not colocalize with the septin cytoskeleton (Supplementary Figure 2). RRAS was detected throughout the cell. Consequently, we were not able to substantiate a significant colocalization with the septins with certainty (Supplementary Figure 2) although we cannot exclude that some colocalizing substructures might be relevant.

In the following, the subcellular distribution of the interaction partners that show substantial co-localization with SEPT9 are discussed in more detail.

For each candidate at least 37 and up to 77 individual cells were monitored. Up to 87% of the evaluated cells showed colocalization of the respective candidate with SEPT9 and the lowest colocalization level was 68%. The values for each considered candidate are shown in Supplementary Table 2.

SNAP23 is part of the SNARE complex (Freedman *et al.* 2003) and involved in docking and fusion of vesicles with their target membrane. It colocalized with SEPT9 at distinct, small dot-like structures at the cell periphery (Figure 4A, VI and Figure 5A, V). In human podocytes it is part of a complex containing SEPT7 and MYH9 (Wasik *et al.* 2017). MYH9, the heavy chain of non-muscle myosin IIA, is not present in our data set. Other septin subunits, namely SEPT5, SEPT7 and SEPT8, have been reported to participate in vesicle docking to the target membrane (Beites *et al*. 2005; Ito *et al.* 2009; Wasik *et al.* 2017). Through SEPT2, septins regulate exocytosis by dynamically interacting with components of the exocytosis apparatus (Tokhtaeva *et al.* 2015). A contribution of SEPT9 in vesicle transport and docking can be anticipated since other constituents of the vesicle trafficking machinery (VAMP2, VABP, COPA, SEC22B) were already identified in a high throughput MS screen as potential SEPT9 interactors (Havugimana *et al.* 2012). Septins have been reported to interact with membrane constituents, to regulate the formation of endocytic carriers at the plasma membrane and to be present on the sorting endosome (reviewed in Song *et al*. 2016). This finding explains why SEPT9 does not colocalize exclusively with filamentous structures inside the cell but appears also as dot- or bar like structures (see Figure 4A or 5A).

For two of the three LIM domain containing candidate proteins, EPLIN and ZNF185, we could show a colocalization with the septins in fibroblast cells by IF. EPLIN colocalized with intracellular SEPT9 on filamentous structures (Figure 4A, VII and Figure 5A, VI), whereas ZNF185 colocalized exclusively with filaments at the cell cortex (Figure 4A, VII and Figure 5B, II). The used antibody for the third protein, LMO7, was unspecific and thus we employed a LMO7-GFP fusion protein to test for colocalization with the endogenous SEPT9. In transiently transfected cells LMO7-GFP colocalized with the septin cytoskeleton (Figure 4B). LMO7 is reported to bind the nuclear membrane protein EMERIN which is a key player in Emery–Dreifuss muscular dystrophy. Moreover, it shuttles between the plasma membrane and the nucleus (Holaska, Rais-Bahrami, and Wilson 2006) and seems to control mitosis progression and exerts an effect on the spindle assembly checkpoint (Tzeng *et al.* 2018). LMO7 is also localized at focal adhesions (Wozniak *et al.* 2013). Through the outcome of a high throughput interaction screen, LMO7 was linked to the actin cytoskeleton by its potential interaction with different myosins (including MYO1C), anillin, beta-actin and EPLIN (Hein *et al.* 2015). EPLIN in turn is a regulator of actin dynamics by bundling actin filaments (Collins *et al.* 2015) and provides a direct physical link between the actin cytoskeleton, the cadherin–catenin complex in adherens junctions (Abe and Takeichi 2008) and the septins (see above). EPLIN interacts with CAV1 (Ohoka *et al.* 2015), one of our Class I SEPT9 interactors that eluded from IF validation due to the non-specificity of the used antibody.

ZNF185 was previously found to interact with F-actin and to be enriched in focal adhesions (Zhang *et al*. 2007). Adherens junctions and focal adhesions use actin for cytoskeletal attachment whereas desmosomes and hemidesmosomes are attached via intermediate filaments. Adherens junctions and focal adhesions connect cells with other cells or with the extracellular matrix, respectively (Maruthamuthu *et al.* 2010). LMO7, EPLIN and ZNF185 serve as intracellular adaptor proteins in these cell junction systems.

Septins associate with the distal ends of radial stress fibers anchored to focal adhesions. It is supposed that septins mediate the anchoring of radial to transverse arc stress fibers. If this event occurs between fibers of opposing focal adhesions, septins may promote the generation of ventral stress fibers (summarized in (Spiliotis 2018)).

We stained ZNF185 and Vinculin, a focal adhesion protein, simultaneously in GFP-SEPT9 expressing cells (Figure 5C). ZNF185 colocalizes with SEPT9-decorated septin filaments as seen before and is enriched in focal adhesions. The focal adhesions seem to be connected by the septin decorated filaments but SEPT9 does not colocalize inside the adhesion complexes. Taken together, these findings support our assumption that anchoring of the septins to the focal adhesion associated fibers is mediated by ZNF185.

Myosin phosphatase targeting subunit (MYPT1) is a non-catalytic subunit of myosin phosphatase and participates in cytoskeleton organization, cell migration and cytokinesis (Matsumura and Hartshorne 2008). It is located along stress fibers in fibroblasts and also to cell adhesions (Murata *et al.* 1997; Inagaki *et al.* 1997). In a high throughput screen, MYPT1 was found as presumable SEPT9 interactor (Hein *et al.* 2015). In our cell culture model, MYPT1 colocalizes with SEPT9 decorated filaments spanning almost the whole length of the cell (Figure 4A, IV and 5A, III).

Another protein interacting with stress fiber associated myosin phosphatase is MPRIP. This interaction is suggested to participate in the recruitment of myosin phosphatase to dephosphorylate myosin light chains on stress fibers (Surks *et al*. 2005). As we found SEPT9 colocalizing with both MYPT1 and MPRIP (Figure 4A, V and 5A, IV), we suggest that the septins serve in this context as a scaffold that bring myosin phosphatase, its regulatory subunits and its intracellular substrates in close proximity.

Apart from these myosin regulatory proteins, we could show that three myosin motors, MYO1C, MYH10 and MYO6, colocalized with the septin cytoskeleton. All are unconventional, non-muscle myosins and are responsible for different aspects in intracellular transport pathways. Type I myosins (among these MYO1C and MYO1B) act as linkers between the actin cytoskeleton and membranes during exocytosis (Kittelberger *et al.* 2016). MYO1C was previously found to be required for the insulin stimulated, actin dependent translocation of GLUT4 containing vesicles toward the plasma membrane (Bose *et al.* 2002). A similar context was reported for MYH9 in podocyte cells. Here, GLUT4 vesicle docking was found to be dependent on a complex built of MYH9, SNAP23 and SEPT7 (Wasik *et al.* 2017). As we have identified SNAP23 as SEPT9 interactor (see above), we speculate that a similar complex consisting of MYO1C, SNAP23 and SEPT9 exists in fibroblasts. GLUT4 is expressed in dermal fibroblast cells (Longo *et al.* 1990) and MYO1C colocalized with SEPT9 at the cell cortex (Figure 4A, I and 5A, I).

MYO6 is an unconventional myosin that moves toward the minus end of actin filaments (Wells *et al.* 1999). It colocalized with SEPT9 as dotted, intracellular structures or with bend filaments below the cortex (Figure 4A, II and 5B, I). MYO6 is involved in targeted membrane transport during cytokinesis (Arden *et al.* 2007) and in the transport of uncoated endocytic vesicles from the actin rich cell periphery toward the endosome (Aschenbrenner *et al.* 2003). Septins were shown to regulate the formation of endocytic carriers at the plasma membrane and to participate in endosomal sorting (reviewed in (Song *et al*. 2016)). These functions might be supported by the interaction of SEPT9 with MYO6.

Are interactions of MYO6 and MYO1C with SEPT9 direct or mediated by adapter proteins? To answer this question, we constructed GST fusion proteins of C-terminal tail fragments of these two myosins. Both fragments cover the helical- and cargo binding domains but not the IQ motifs and the motor domains. A similar fragment of MYO6 was previously used for GST pulldown experiments (Wollscheid *et al.* 2016). The MYO1C fragment was prepared correspondingly. We performed a pulldown with recombinantly expressed, 6his tagged SEPT9. SEPT9 specifically interacts with both GST-MYO6_835-1294_ and GST-MYO1C_809-1063_ *in vitro* (Figure 5D).

Another septin, SEPT2, is reported to interact directly with myosin motors, particularly with a tail fragment of non-muscle myosin IIA containing the myosin heavy chain MYH9 (Joo *et al*. 2007). MYH9 was not identified as interactor of SEPT9 in our screen but the heavy chain of myosin IIB, MYH10, showed a colocalization with SEPT9 decorated filaments (Figure 4A, III and 5A, II). We propose an isoform specific interaction with certain septin subunits - in this case myosin IIA with SEPT2 and myosin IIB with SEPT9.

The interaction of SEPT2 with MYH9 was found to be important for the stability of stress fibers and loss of the interaction caused instability of the ingressed cleavage furrow in dividing cells (Joo, Surka, and Trimble 2007). The direct interaction of SEPT9 with myosin motors point toward a similar role of SEPT9 especially in vesicle docking during exocytosis and endocytosis.

Taken together, we present a list of novel SEPT9 interacting proteins and validated a subset of candidate proteins by IF and pull-down experiments. Especially the involvement of SEPT9 in stress fiber anchoring to adherens junctions and the direct interaction with the cargo domains of myosin motors opens new perspectives for the role of the septins in intracellular processes.
